# Endovascular Treatment of Atherosclerotic Tandem Occlusions in Anterior Circulation Stroke: Technical Aspects and Complications Compared to Isolated Intracranial Occlusions

**DOI:** 10.3389/fneur.2018.01046

**Published:** 2018-12-13

**Authors:** Omer Faruk Eker, Monika Bühlmann, Cyril Dargazanli, Johannes Kaesmacher, Isabelle Mourand, Jan Gralla, Caroline Arquizan, Urs Martin Fischer, Gregory Gascou, Mirjam Heldner, Marcel Arnold, Vincent Costalat, Pasquale Mordasini

**Affiliations:** ^1^Department of Interventional and Diagnostic Neuroradiology, Montpellier University Hospital, Montpellier, France; ^2^Department of Neurology, Inselspital, Bern University Hospital, Bern, Switzerland; ^3^University Institute of Diagnostic and Interventional Neuroradiology, Inselspital, Bern University Hospital, Bern, Switzerland; ^4^Department of Neurology, Montpellier University Hospital, Montpellier, France

**Keywords:** stroke, endovascular treatment, tandem occlusion, thrombectomy, stenting

## Abstract

**Background and Purpose:** Endovascular treatment of tandem occlusions is an emerging option. We describe our multicenter experience with endovascular management of atherosclerotic tandem occlusions in the anterior circulation, particularly the technical aspects and complications in comparison to isolated intracranial occlusions.

**Materials and Methods:** Consecutive patients with tandem occlusions due to atherosclerotic causes who underwent mechanical thrombectomy at two major stroke centers between January 2010 and September 2015 were reviewed. Clinical data, procedural aspects, recanalization rates, complication rates, and clinical outcome were analyzed and compared to findings in patients with isolated intracranial occlusions.

**Results:** One hundred and twenty-one patients with tandem occlusions and 456 patients with isolated intracranial occlusions (carotid-T/M1) were included. Mean intervention time was faster (33 min vs. 57 min, *p* < 0.001) and recanalization success was higher (TICI 2b/3 83.6 vs. 70.2%, *p* = 0.002) in patients with isolated occlusions. No difference was seen in clinical outcome and complications, except for a higher rate of asymptomatic hemorrhage in the tandem group (29.8 vs. 17.1%, *p* = 0.003). Choice of recanalization approach (antegrade vs. retrograde) in the tandem group made no difference, except for a trend toward less distal emboli using the retrograde approach (4.0 vs. 13.0%, *p* = 0.082). Stenting of the extracranial internal carotid artery (ICA) was performed in 81%, PTA alone in 7.4%, and deferred stenting in 11.6%. Rate of stent/ICA occlusion within 7 days was 10.3% after stenting and 33.3% after PTA (*p* = 0.127). In the tandem group, age (*p* = 0.034), National Institutes of Health Stroke Scale score (NIHSS) at admission (*p* = 0.002), recanalization rate (*p* < 0.001), complications (*p* = 0.016), and symptomatic intracranial hemorrhage (sICH) (*p* = 0.001) were associated with poor outcome, whereas extracranial treatment modality and stent/ICA occlusion within 7 days did not affect outcome.

**Conclusion:** Endovascular treatment of tandem occlusions is technically feasible, achieves recanalization rates and rates of good clinical outcome comparable to those in patients with isolated intracranial occlusions. Following acute ICA stenting, the risk of stent occlusion and sICH appeared to be low, but was associated with an increased rate of asymptomatic ICH.

## Introduction

Acute tandem occlusions of the extracranial internal carotid artery (ICA) due to an atherosclerotic lesion combined with an intracranial ICA/middle cerebral artery (MCA) occlusion cause a severe form of ischemic stroke associated with high rates of disability and death. Endovascular treatment with stent retrievers, with or without concomitant extracranial stenting of the underlying arteriosclerotic lesion, is an emerging treatment option for this complex form of ischemic stroke (1–8). However, endovascular treatment is challenging and optimal treatment strategies to enhance recanalization rates and to avoid peri-procedural complications, thus improving clinical outcome, are still uncertain.

Several recent randomized-controlled trials have shown the superiority of endovascular treatment of large vessel occlusions in acute ischemic stroke of the anterior circulation using stent-retriever thrombectomy over intravenous thrombolysis (9–13). Due to the greater severity of this form of stroke and the technical complexity of endovascular treatment of tandem occlusions, this subgroup of stroke patients has been systematically excluded from most of the randomized-controlled trials. Endovascular recanalization in these patients is technically feasible, but is more complex than in those with isolated intracranial large vessel occlusion (1, 8, 14–16).

Therefore, consensus has yet to be reached on optimal treatment strategies to maximize recanalization success while minimizing treatment-related complications, such as distal cerebral thromboembolic events, stent occlusion, and symptomatic intracranial hemorrhage (sICH).

The purpose of the present multicenter study was to compare stroke patients with acute tandem occlusions due to an atherosclerotic cervical lesion and patients with isolated carotid-T/M1 occlusions undergoing endovascular recanalization. Comparisons were made of clinical and imaging data, procedural aspects, recanalization rates, complication rates, and clinical outcome. In addition, we reviewed our experience of the endovascular management of atherosclerotic tandem occlusions of the anterior circulation paying special regard to technical and peri-procedural aspects.

## Materials and Methods

### Patient Selection

Records of all patients presenting with anterior circulation ischemic stroke who were treated with an endovascular approach at two separate institutions between January 2010 and September 2015 were retrospectively retrieved from prospectively maintained stroke databases. The registry was approved by the local ethics committee (Kantonale Ethikkommission für die Forschung Bern, Bern, Switzerland, amendment access number: 231/2014) and included baseline patients characteristics, risk factors, time metrics, and recorded clinical data). Consecutive patients with extra-intracranial tandem occlusions due to an atherosclerotic cervical lesion who underwent mechanical stent-retriever thrombectomy with or without extracranial stent implantation and patients with isolated occlusions of the anterior circulation (i.e., carotid-T and M1 segments of the MCA) were reviewed and included in this study.

Endovascular therapy was performed immediately after computed tomography (CT) or magnetic resonance imaging (MRI) if: (1) diagnosis of ischemic stroke was established by CT/CT angiography (CTA)/CT perfusion or MRI/MR angiography (MRA)/MR perfusion imaging; (2) baseline National Institutes of Health Stroke Scale (NIHSS) score was ≥4 or isolated aphasia or hemianopia was present; (3) hemorrhage had been excluded by cranial CT or MRI; (4) symptom duration was not longer than 24 h; (5) no clinical or premorbid conditions or laboratory findings contraindicated treatment; (6) isolated intracranial large vessel occlusion or tandem occlusion of the carotid-T or M1 segment associated with cervical ICA obstruction or occlusion related to ICA atheromatosis was demonstrated by initial imaging and/or peri-interventional angiograms.

### Decision-Making and Imaging

Initial NIHSS was assessed by a dedicated stroke neurologist. CT and MRI were used according to the center's protocol. The routine CT imaging protocol consisted of unenhanced CT scan and CTA as well as CT perfusion. The routine MRI protocol consisted of diffusion weighted imaging (DWI), fluid-attenuated inversion recovery (FLAIR), T2^*^/susceptibility-weighted imaging (SWI), contrast-enhanced MR angiography including the supra-aortic trunks, and MR perfusion imaging. If tandem occlusion was suspected from initial imaging, it was confirmed with digital subtraction angiography (DSA) based on the morphological aspect of the cervical segment of the internal carotid artery (ICA) associated with a proximal intracranial vessel occlusion (carotid termination, M1 segment). Diagnosis of atherosclerotic lesions was based on clinical, imaging, and angiographic data. In particular, we aimed to distinguish dissection from atheromatous cervical carotid artery lesions.

### Intravenous Thrombolysis (IVT)

IVT (0.9 mg/kg; 10% of the dose as a bolus and the remainder over 60 min) was administered to patients within a maximum of 4.5 h after stroke onset. Conventional clinical and laboratory inclusion and exclusion criteria for IVT were applied.

### Endovascular Procedure

Our general technical approach to address tandem lesions in acute ischemic stroke is described elsewere (17). In brief, endovascular treatment was performed under general anesthesia or conscious sedation. The decision on type of sedation was made on an individual basis by the neurologist, -neuroradiologist, and anesthesiologist on call. DSA was performed via a transfemoral approach using a biplane, high-resolution angiography system. An 8 French guiding catheter or an 8 or 9 French balloon-guiding catheter (Guider Softip XF, Boston Scientific, Marlborough, USA; Merci, Concentric Medical, Mountain View, USA) was introduced into the common carotid artery and an angiographic run was performed to evaluate the occlusion. During this step, cervical ICA occlusion/stenosis was demonstrated and eventually differentiated from carotid contrast agent stagnation related to isolated carotid termination thrombus. Taking into account both clinical data and angiographic morphology, atheromatous occlusion was distinguished from ICA dissection.

In general, a retrograde revascularization approach (distal-to-proximal approach), i.e., first, recanalization of the intracranial occlusion by mechanical thrombectomy after passing through the stenosis and secondly, addressing the cervical ICA lesion, was used whenever primary passing of the atheromatous lesion was technically feasible. A 0.021 “microcatheter (Headway, MicroVention Terumo, Aliso Viejo, USA; Prowler Select Plus, Codman & Shurtleff, Raynham, USA) was navigated through the stenosis over a 0.014” microwire (Transend 014, Boston Scientific, Marlborough, USA; Traxcess, MicroVention, Aliso Viejo, USA; Silverspeed-14, ev3, Irvine, USA). This maneuver was performed under flow arrest either through wedging of the guide catheter in the stenosis, or inflation of the balloon-guide catheter to avoid thromboembolic events during crossing of the stenosis. Once the microcatheter position distally had been confirmed by a careful contrast injection, an intermediate 5 or 6 French guiding catheter (5max and 5max ACE, Penumbra, Alameda, USA; Vasco+35Aspi, Balt Extrusion, Montmorency, France; Sophia 5/6F, Microvention, Aliso Viejo, USA) was then advanced over the microcatheter into the distal ICA. After crossing the intracranial thrombus over the microwire with the microcatheter, mechanical thrombectomy was performed with a Solitaire FR (ev3, Irvine, USA) or Trevo (Stryker Neurovascular, Mountain View, USA). The thrombectomy maneuver was also performed under flow arrest with manual aspiration through the intermediate catheter to prevent clot fragmentation and distal embolism. The result of intracranial recanalization was evaluated using the thrombolysis in cerebral infarction (TICI) score. Successful recanalization was defined as TICI 2b or 3. If intracranial recanalization was achieved, treatment of the cervical carotid stenosis was left to the discretion of the operator, taking into account residual cervical occlusion/severe stenosis or thrombus apposition.

To perform cervical stenting or PTA, the 0.014“ microwire was introduced into the petrous segment of the carotid artery to preserve distal access. At one center, a distal filter protection device (FilterWire EZ, Boston Scientific, Marlborough, USA) was placed distal to the stenosis when anatomically possible. Then, the guiding catheter and the intermediate catheter were retrieved into the common carotid artery. A 250 mg bolus of aspirin was administered intravenously prior to stenting. The decision to introduce double anti-platelet therapy with 75 mg clopidogrel was taken following a control CT scan within 24 h, depending on the presence of hemorrhagic transformation or intracerebral hemorrhage. Cervical carotid artery stenting was performed (Carotid Wallstent, Boston Scientific, Natick, USA; Precise, Codman and Shurtleff, Raynham, USA; Cristallo ideale, Invatec, Roncadelle, Italy; Casper, Microvention, Tustin, USA; X-ACT, Abbott Vascular, Redwood, USA). Appropriate positioning and opening of the stent was immediately assessed angiographically and additional angioplasty was performed if deemed necessary. In PTA-only cases, PTA was performed using different PTA balloons (Submarine Rapido, Invatec, Roncadelle, Italy; Aviator Plus, Cordis, Freemont, USA; Ultrasoft balloon dilatation catheter, Boston Scientific, Freemont,USA).

If initial passing through the stenosis with the guiding or intermediate catheter was not possible, an antegrade (proximal-to-distal) approach was used, i.e., first addressing the ICA stenosis with PTA/stenting under proximal flow arrest using a balloon-guiding catheter and/or a distal filter protection device and then performing the intracranial thrombectomy as the second step as described above.

In one center, in case of an efficient Circle of Willis and no threatening cervical steno-occlusive lesion, the treatment of the latter was deferred to the subacute phase (after 2–5 days) on a semi-elective basis after initiating double anti-platelet therapy.

### Follow-Up

Follow-up CT or MRI was performed 24 h after the acute therapy to assess infarction volume and hemorrhagic status. Symptomatic intracranial hemorrhage (sICH) was defined as a documented hemorrhage associated with a decline of ≥4 points in the NIHSS score. If no hemorrhage occurred, double anti-platelet therapy was initiated and continued for 3–6 months. Single anti-platelet treatment was then maintained for 1 year or lifelong depending on the responsible center's protocol.

NIHSS was measured following recovery from the anesthetic, and throughout hospitalization until discharge. Routine clinical follow-up was performed at 3 months by an independent neurologist to evaluate patients' recovery, using the mRS. Clinical outcome was quantified by 3-months mRS and mortality. Favorable outcome was defined as mRS ≤ 2.

### Statistical Analysis

Group comparison of continuous or ordinally scaled variables was carried out with the Mann-Whitney-U or Kruskal-Wallis test depending on the number of groups. Frequency counts were compared by applying Fisher's exact test. For multivariable logistic regression analysis, all variables with *p* < 0.150 in the univariate analyses were included in the model. Analyses were run using IBM SPSS Statistics v.21 (IBM, Armonk, NY, USA).

## Results

### Clinical Baseline, Interventional and Outcome Results in Tandem Occlusions vs. Isolated Intracranial Occlusions

Between January 2010 and September 2015, 121 patients with atherosclerotic tandem occlusions and 456 patients with isolated carotid-T or M1 occlusions were consecutively treated in the two participating institutions using mechanical thrombectomy with stent-retrievers. Patients in the isolated intracranial occlusion group were predominantly female (50.4 vs. 25.6%, *p* < 0.001). Patients in the tandem occlusion group were more likely to have a carotid-T occlusion (32.2 vs. 19.1%, *p* = 0.003) and to be smokers (*p* < 0.001). Patients in the isolated intracranial occlusion group had a higher prevalence of atrial fibrillation (*p* = 0.003). There was no difference between the two groups in NIHSS score at admission (17, interquartile range [IQR] 12–20 vs. 16, IQR 13–21, *p* = 0.474) and time from symptom-onset to groin puncture (262 min, IQR 194–356 vs. 241, IQR 190–315, *p* = 0.093). Procedure time was significantly shorter in the group with isolated intracranial occlusion (33 min, IQR 49–89 vs. 57 min, IQR 87–115, *p* < 0.001) and successful recanalization (TICI 2b/3) was more frequent (83.6 vs. 70.2%, *p* = 0.002). There was no difference in technical complication rate, including infarcts to previously unaffected vessel territories. The rate of asymptomatic ICH was higher in the tandem group (29.8 vs. 17.1%, *p* = 0.003); however there was no significant difference in the rate of sICH (10.7 vs. 6.9%, *p* = 0.177). Rate of good outcome (mRS ≤ 2) and mortality at 90 days was similar in the tandem group and the isolated intracranial occlusion group, (42.4 vs. 59.6%, *p* = 0.177; and 19.5 vs. 15.7%, *p* = 0.329, respectively). Clinical baseline, interventional and outcome characteristics are summarized in Table 1.

**Table 1:** Clinical baseline, interventional and outcome characteristics.

	**Tandem intracranial-extracranial occlusions (*N* = 121)**	**Isolated intracranial occlusions (*N* = 456)**	***P***
**BASELINE**			
Age (years)	72 (61–79)	70 (63–78)	0.544
Sex, female	25.6% (31/121)	50.4% (230/456)	< 0.001
Admission NIHSS	17 (IQR 12–20)	16 (IQR 13–21)	0.474
IVT	52.1% (63/121)	53.9% (246/456)	0.759
Intracranial occlusion site			0.003
- M1	67.8% (82/121)	80.9% (369/456)
- ICA	32.2% (39/121)	19.1% (87/456)
Symptom-onset to groin puncture (mins)	262 (IQR 194–356)	241 (IQR 190–315)	0.093
Atrial fibrillation	30.0% (36/120)	45.2% (192/425)	0.003
Diabetes	20.7% (25/121)	13.6% (62/455)	0.063
Arterial hypertension	62.8% (76/121)	65.2% (296/454)	0.669
Dyslipidemia	51.3% (61/119)	45.9% (208/453)	0.304
Smoking	45.8% (54/118)	24.2% (102/421)	< 0.001
**INTERVENTIONAL**			
Procedure time	57 (IQR 87–115)	33 (IQR 49–89)	< 0.001
Number of maneuvers	1 (IQR 1–3)	1 (IQR 1–3)	0.495
Final TICI 2b/3	70.2% (85/121)	83.6% (381/456)	0.002
Complications			0.122
- None - Dissection - Perforation - Other	- 89.3% (108/121) - 7.4% (9/121) - 2.5% (3/121) - 0.8% (1/121)	- 93.2% (425/456) - 5.7% (26/456) - 0.4% (2/456) - 0.7% (3/456)
Infarct in previously unaffected territories	7.4% (9/121)	8.6% (39/456)	0.853
**OUTCOME**			
sICH	10.7% (13/121)	6.9% (31/452)	0.177
aICH	29.8% (36/121)	17.09% (77/452)	0.003
90-day mRS	3 (IQR 1–4)	3 (IQR 1–4)	0.142
90-day mRS ≤ 2	42.4% (50/118)	49.6% (212/427)	0.177
90-day mortality	19.5% (23/118)	15.7% (67/427)	0.329

### Results of Management Strategies, Technical and Clinical Outcome in the Tandem Occlusion Group

Most of the patients in the tandem occlusion group were treated using the retrograde approach 62% (75/121). There was no difference in technical or clinical outcome related to recanalization approach, except for a trend toward fewer distal emboli when the retrograde approach was used (4.0 vs. 13.0%, *p* = 0.082). Stenting of the extracranial ICA was performed in 81% of patients (98/121), PTA alone in 7.4% (9/121), and deferred stenting in 11.6% (14/121). There was a statistically non-significant trend toward a higher rate of stent/ICA occlusion within 7 days after PTA-only compared to stenting (33.3 vs. 10.3%, *p* = 0.127).

In the tandem occlusion group, age (*p* = 0.034), NIHSS at admission (*p* = 0.002), recanalization rate (*p* < 0.001), any complication (*p* = 0.016), and sICH (*p* = 0.001) were associated with poor outcome, whereas extracranial treatment modality (stenting, PTA-only, or deferred stenting) and stent or ICA occlusion within 7 days did not affect outcome. In multivariable logistic regression analysis, only NIHSS [aOR 0.89, 95% confidence intervals (CI) 0.82–0.97], age (aOR 0.95, 95% CI 0.91–0.99) and reperfusion success (aOR TICI2b/3 4.77, 95% CI 1.39–16.41) were associated with mRS ≤ 2. Data on the technical and clinical outcome of patients in the tandem occlusion group is summarized in Tables 2–4.

**Table 2:** Interventional and clinical outcome in patients with tandem occlusions treated using the antegrade vs. retrograde approach.

	**Antegrade approach (*n* = 46)**	**Retrograde approach (*n* = 75)**	***P***
**INTERVENTIONAL**			
Age (years)	70 (63–79)	70 (63–76)	0.871
Sex, female	32.6% (15/46)	21.3% (16/31)	0.200
Admission NIHSS	18 (13–21)	16 (10–19)	0.077
Procedure time	95 (IQR 53–141)	82 (IQR 61–109)	0.251
Number of maneuvers	2 (IQR 1–3)	1 (IQR 1–2)	0.017
Intracranial final TICI 2b/3	65.2% (30/46)	73.3% (55/75)	0.414
Complications			0.305
- None - Dissection - Perforation - Other	- 89.1% (41/46) - 10.9% (5/56) - 0% (0/46) - 0% (0/46)	- 89.3% (67/75) - 5.3% (4/75) - 4.0% (3/75) - 1.3% (1/75)
Infarct in previously unaffected territories	13.0% (6/46)	4.0% (3/75)	0.082
sICH	10.9% (5/46)	10.7% (8/75)	1.000
aICH	28.3% (13/46)	30.7% (23/75)	0.840
Rethrombosis during acute treatment	4.3% (2/46)	9.3% (7/75)	0.480
ICA patency at end of procedure	100% (46/46)	97.3% (73/75)	0.525
ICA patency at day 7	93.5% (43/46)	85.1% (63/74)	0.244
90-day mRS	3 (IQR 1–4)	3 (IQR 1–5)	0.300
90-day mRS ≤ 2	46.7% (21/45)	39.7% (29/73)	0.565
90-day mortality	15.6% (7/45)	21.9% (16/73)	0.478

**Table 3:** Interventional outcome in patients with tandem occlusions according to extracranial treatment modality.

	**PTA only (*n* = 9)**	**Acute stenting** **(*n* = 98)**	**Deferred stenting** **(*n* = 14)**	***P***
**INTERVENTIONAL**				
Age, years	70 (IQR 65–86)	71 (IQR 63–78)	67 (IQR 55–76)	0.254
Sex, female	44.4% (4/9)	24.5% (24/98)	21.4% (3/14)	0.377
Admission NIHSS	11 (IQR 7–18)	17 (IQR 12–20)	18 (IQR 15–20)	0.094
Procedure time	71 (IQR 51–121)	92 (IQR 66–120)	75 (IQR 49–95)	0.269
Number of maneuvers	1 (IQR 1–2)	1 (IQR 1–3)	3 (IQR 2–3)	0.048
Intracranial final TICI 2b/3	77.8% (7/9)	69.4% (68/98)	71.4% (10/14)	0.932
Complications				0.398
- None - Dissection - Perforation - Other	- 77.8% (7/9) - 11.1% (1/9) - 11.1% (1/9) - 0% (0/9)	- 88.8% (87/98) - 8.2% (8/98) - 2.0% (2/98) - 0% (0/98)	- 100% (14/14) - 0% (0/14) - 0% (0/14) - 0% (0/14)
Infarct in previously unaffected territories	0% (0/9)	8.2% (8/98)	7.1% (1/14)	1.000
sICH	0% (0/9)	12.2% (12/98)	7.1% (1/14)	0.739
aICH	22.2% (2/9)	31.6% (31/98)	21.4% (3/14)	0.694
Acute in-stent thrombosis during extracranial treatment		9.2% (9/98)	0% (0/14)
ICA patency at end of procedure	100% (9/9)	98.0% (96/98)	100% (14/14)	1.000
ICA occlusion at day 7 after extracranial procedure	33.3% (3/9)	10.3% (10/98)	7.1% (1/14)	0.127
90-day mRS	3 (1–4)	3 (1–5)	2 (0–3)	0.059
90-day mRS ≤ 2	37.5% (3/8)	39.6% (38/96)	64.3% (9/14)	0.231
90-day mortality	0% (0/8)	24.0% (23/96)	0% (0/14)	0.025

**Table 4:** Prognostic factors for good and poor outcome in patients with tandem occlusions.

	**Good outcome** **(90 days mRS ≤ 2,** ***n* = 50)**	**Poor outcome** **(90 days mRS > 2,** ***n* = 68)**	***P***
**BASELINE**			
Age, years	67.2 ± 9.6	71.0 ± 9.5	0.034
Sex, female	26.0% (13/50)	25.0% (17/68)	1.000
Admission NIHSS	15 (10–18)	18 (14–21)	0.002
IVT	44.0% (22/50)	55.9% (38/68)	0.264
Intracranial occlusion site			0.079
- M1	76.0% (38/50)	60.3% (41/68)
- ICA	24.0% (12/50)	39.7% (27/68)
Symptom onset to groin puncture (min)	259 (IQR 193–341)	279 (195–380)	0.365
Atrial fibrillation	26.5% (13/49)	30.9% (21/68)	0.682
Diabetes	14.0% (7/50)	25.0% (17/68)	0.170
Arterial hypertension	56.0% (28/50)	66.2% (45/68)	0.338
Dyslipidemia	54.2% (26/48)	50.0% (34/68)	0.708
Smoking	52.0% (26/50)	43.1% (28/65)	0.354
**INTERVENTIONAL**			
Procedure time	74 (IQR 55–108)	94 (IQR 71–120)	0.122
Number of maneuvers	1 (IQR 1–2)	2 (IQR 1–3)	0.193
Final TICI 2b/3	88.0% (44/50)	55.9% (38/68)	<0.001
Antegrade	42.0% (21/50)	35.3% (25/68)	0.565
Extracranial treatment modality			0.231
- Acute stenting ± PTA	76.0% (38/50)	85.3% (58/68)
- PTA only	6.0% (3/50)	7.4% (5/68)
- Deferred stenting ± PTA	18.0% (9/14)	7.4% (5/68)
ICA patency after 7 days	90.0% (45/50)	86.6% (58/67)	0.775
Any complications	8.0% (4/50)	26.5% (18/68)	0.016
sICH	0% (0/50)	19.1% (13/68)	0.001
aICH	28.0% (14/50)	30.9% (21/68)	0.839

## Discussion

In patients with acute ischemic stroke, rapid recanalization has been shown to have a strong influence on favorable clinical outcome. Several recent randomized-controlled trials have shown the superiority of endovascular mechanical thrombectomy with stent-retrievers in large vessel occlusions in acute ischemic stroke of the anterior circulation over intravenous thrombolysis, achieving higher recanalization rates and higher rates of good clinical outcome (9–11, 13, 18). However, approximately 20% of patients with an intracranial large vessel occlusion of the anterior circulation have an additional high-grade extracranial ICA stenosis or occlusion. Stenotic or occlusive lesions of the ICA may be caused by atherosclerotic disease, acute dissection or large thromboemboli. These lesions are a major potential cause of intracranial embolism in the carotid-T or the MCA as well as additional concomitant hemodynamic impairment of brain perfusion. This is associated with a risk of developing a large MCA infarction with morbidity rates up to 70% and mortality rates up to 55% (19). Owing to the large clot burden and/or slow distal flow, the combination of extracranial occlusive disease and intracranial occlusion has shown a poor response to IVT with recanalization rates only around 20% and poor outcome in up to 80–100% (20). Therefore, endovascular treatment of tandem occlusions with stent-retrievers with or without concomitant extracranial PTA/stenting of the underlying obstructive lesion is an emerging treatment option for this complex form of ischemic stroke in order to achieve faster and higher rates of recanalization. However, due to the greater severity of this form of stroke and the technical complexity of endovascular treatment of tandem occlusions, this subgroup of stroke patients has been systematically excluded from most of the prospective randomized-controlled trials. Several published studies have shown that endovascular recanalization is technically feasible, but complex. Successful recanalization rates have been achieved in 60–80% of patients and a wide range of good clinical outcomes from 39 to 63% has been reported (1–8, 14–16, 21). In our study, rates of favorable clinical outcome and mortality were similar in acute stroke patients with tandem lesions to those with isolated intracranial large vessel occlusions. This emphasizes that these patients have the chance of a similar clinical outcome and endovascular treatment should be considered the standard of care (22, 23).

Notably, the rate of successful recanalization (TICI ≥ 2B) was lower in the tandem lesion group. It was, however, still within the range reported in the literature for patients with isolated large vessel occlusion of the anterior circulation treated with mechanical thrombectomy using stent-retrievers (9–11, 13, 18). Overall, technical complications, including distal emboli in previously unaffected territories and sICH, were similar despite the more complex technical approaches needed for the tandem occlusion group confirming a comparable overall safety profile.

### Recanalization Strategies: Antegrade vs. Retrograde Approach

Two different recanalization strategies have been proposed: The “antegrade approach” (also called the proximal-to-distal or extracranial-first approach) consists of first addressing the extracranial carotid lesion using PTA and/or stenting and recanalization of the intracranial occlusion using mechanical thrombectomy second. This strategy may allow for safer access to the intracranial lesion, better control of distal thromboemboli, and may lower the risk of distal re-occlusion due to the re-established and increased antegrade flow (3, 15, 24–28). However, recanalization of the extracranial carotid lesion before treatment of the distal intracranial lesion can be time-consuming and can delay intracranial recanalization, potentially leading to a less favorable outcome. Thus, the “retrograde approach” (also known as the distal-to-proximal or intracranial-first approach) aims at recanalization of the distal and symptomatic intracranial occlusion first (3, 4, 17, 24, 25, 29–31). Rapid restoration of perfusion to the affected territory first, by distal recanalization using thrombectomy, shortens ischemic time and may increase the likelihood of a favorable outcome. Once the distal occlusion has been recanalized, time can be spent on definitively addressing the carotid occlusive lesion. The major disadvantage of this approach is that treating the carotid lesion after recanalization of the distal occlusion may expose the patient to the potential risk of distal embolization in the intracranial vasculature. Therefore, additional protective measures such as the use of distal filter protection devices or proximal balloon occlusion during PTA/stenting have been advocated. In our study, the preferred recanalization strategy was the retrograde approach. However, the type of approach used did not affect procedure time, recanalization success or clinical outcome. Furthermore, there was a statistically non-significant trend toward a higher incidence of distal emboli using the antegrade approach. This might be explained by the consequent flow arrest achieved during intracranial thrombectomy using the retrograde approach and proximal and/or simultaneous distal protection when addressing the ICA stenotic lesion. These findings are in line with a recent meta-analysis of the management of tandem occlusions in patients with acute ischemic stroke (8). No differences were observed in technical and clinical outcomes with respect to the recanalization strategy except for a trend toward a higher rate of procedure-related complications in patients treated with the antegrade approach (20 vs. 8%, *p* = 0.25).

### Extracranial Treatment Modalities and Anti-platelet Therapy

Different extracranial treatment techniques have been advocated to minimize the risk of re-occlusion, re-embolization, and neurological deterioration related to hemodynamic impairment due to symptomatic ICA stenosis. Stenting in the acute phase enables the interventionalist to secure and protect a symptomatic plaque and thus decrease the risk of recurrence of stroke and re-establish flow in the case of hemodynamic impairment allowing for a definitive, “one-stop” treatment of symptomatic ICA stenosis. On the other hand, a PTA-only strategy also enables treatment of hemodynamic impairment without the need for anti-platelet therapy and deferred treatment by carotid endarterectomy or stenting under “optimal” conditions (28, 29, 32, 33).

If carotid artery stent placement is necessary, anti-thrombotic medication has to be administered to prevent acute in-stent thrombosis carrying the risk of *de-novo* distal thromboembolization or stent occlusion. However, giving any kind of anti-thrombotic medication to patients with acute stroke increases the risk of ICH (34, 35). Widely differing anti-thrombotic treatment strategies are described in the literature. The reported anti-thrombotic regimen includes administration of aspirin alone or in combination with clopidogrel at various dosages as well as the administration of glycoprotein IIb/IIIa inhibitors and heparin. Rates of sICH of up to 30% have been observed and anti-thrombotic treatment has been identified as a major potential source of complications (34–36). In the present study, anti-platelet monotherapy with 250 mg of aspirin was administered in the acute phase resulting in a similar rate of sICH in the tandem lesion group to that in the group with isolated occlusions, but with a higher rate of asymptomatic ICH. These findings are in line with recently reported rates of sICH in patients treated for tandem occlusions (5, 6, 8, 22, 36). Furthermore, anti-platelet therapy with aspirin only after bridging IVT did not lead to any increase in sICH or have a negative effect on clinical outcome, a finding which is in line with previously published data (34, 37, 38).

The risk of sICH after stenting in the acute phase has to be balanced against the risk of stent occlusion or thrombosis and its clinical consequences. As yet data on stent occlusion within the first few days or its clinical relevance is scarce. In this series, the rate of stent/ICA occlusion within 7 days after stenting in the acute phase was 10.3% with a trend toward higher rates (33.3%) after PTA-only. Interestingly, stent/ICA occlusion *per se* was not associated with worse outcome and all affected patients remained clinically asymptomatic. This might be due the long-standing character of the atherosclerotic ICA stenosis leading eventually to better hemodynamic tolerance of the occlusion without further distal embolic events. Overall, technical complications did not differ between the extracranial treatment modalities, which is in agreement with previously reported data (8).

The results of our study suggest that anti-platelet therapy with aspirin only in the acute phase of stroke does not significantly increase the rate of sICH and has no overall negative impact on clinical outcome irrespective of previously administered bridging IVT. On the other hand, approximately 10% of stents showed early occlusion, although in our series there were no clinical consequences in terms of recurrence of stroke. Therefore, stenting in the acute phase with aspirin only as an anti-platelet therapy seems to yield an acceptable risk–benefit ratio in most cases regarding rate of sICH and of stent occlusion.

### Limitations

This study has inherent limitations due to the retrospective design, although the data is derived from a prospectively collected database. Moreover, the application of different multimodal approaches for thrombectomy in the setting of atherosclerotic tandem occlusion might confound the treatment effect. Furthermore, the PTA-only and deferred-stenting subgroups were significantly smaller than the stenting group, limiting the comparability of the results. Finally, definitive conclusions about the management of tandem occlusions cannot be drawn from this study and further clinical studies, preferably in the setting of a multicenter randomized-controlled trial, are warranted, e. g. comparing safety, technique and outcome of immediate vs. deferred stenting.

## Conclusions

Endovascular treatment of tandem occlusions is technically feasible, achieves recanalization rates and rates of good clinical outcome comparable to those for isolated intracranial occlusions and should therefore be considered to be the standard of care. In cases of acute ICA stenting, the risk of stent occlusion and sICH seems to be acceptable although it was associated with an increased rate of asymptomatic ICH due to the need for anti-platelet therapy.

## Ethics Statement

This study was carried out in accordance with the recommendations of name of guidelines, name of committee with written informed consent from all subjects. All subjects gave written informed consent in accordance with the Declaration of Helsinki. The protocol was approved by the name of committee.

## Author Contributions

OFE, MB, VC, and PM conceived and designed the study. OFE, MB, and MH acquired the data. OFE, MB, JK, VC, and PM analyzed and interpreted the data. JK and PM performed statistical analysis. OFE, MB, VC, and PM drafted the manuscript. OFE, MB, CD, JK, IM, JG, CA, UF, GG, MA, VC, and PM made critical revisions of the manuscript.

### Conflict of Interest Statement

OE is a consultant for Stryker and his institution receives payment for educational presentations by Covidien, Medtronic, and Stryker. JG is a global principal investigator of STAR (Solitaire FR Thrombectomy for Acute Revascularisation), clinical event committee member of the PROMISE study (European Registry on the ACE Reperfusion Catheters and the Penumbra System in the Treatment of Acute Ischemic Stroke; Penumbra), and a principal investigator and consultant for the SWIFT DIRECT study (Medtronic) and receives Swiss National Science Foundation (SNSF) grants for magnetic resonance imaging in stroke. UF is a global principal investigator for the SWIFT DIRECT study (Medtronic) and receives research grants from the SNSF. MA has received speaker honoraria from Amgen, Bayer, Boehringer Ingelheim (BI), Medtronic and Covidien, and scientific advisory board honoraria from Bayer, BI, BMS, Pfizer, Covidien, Daichy Sankyo and Nestlé Health Science. The remaining authors declare that the research was conducted in the absence of any commercial or financial relationships that could be construed as a potential conflict of interest.
